# Interacting impact of maternal inflammatory response and stress on the amygdala transcriptome of pigs

**DOI:** 10.1093/g3journal/jkab113

**Published:** 2021-04-15

**Authors:** Marissa R Keever-Keigher, Pan Zhang, Courtni R Bolt, Haley E Rymut, Adrienne M Antonson, Megan P Caputo, Alexandra K Houser, Alvaro G Hernandez, Bruce R Southey, Laurie A Rund, Rodney W Johnson, Sandra L Rodriguez-Zas

**Affiliations:** 1 Department of Animal Sciences, University of Illinois at Urbana-Champaign, Urbana, IL 61820, USA; 2 Illinois Informatics Institute, University of Illinois at Urbana-Champaign, Urbana, IL 61820, USA; 3 High-Throughput Sequencing and Genotyping Unit, Roy J. Carver Biotechnology Center, University of Illinois at Urbana-Champaign, Urbana, IL 61820, USA; 4 Neuroscience Program, University of Illinois at Urbana-Champaign, Urbana, IL 61820, USA; 5 Department of Statistics, University of Illinois at Urbana-Champaign, Urbana, IL 61820, USA; 6 Carl R. Woese Institute for Genomic Biology, University of Illinois at Urbana-Champaign, Urbana, IL 61820, USA

**Keywords:** RNA-seq, gestational stress, weaning stress, double-hit hypothesis

## Abstract

Changes at the molecular level capacitate the plasticity displayed by the brain in response to stress stimuli. Weaning stress can trigger molecular changes that influence the physiology of the offspring. Likewise, maternal immune activation (MIA) during gestation has been associated with behavior disorders and molecular changes in the amygdala of the offspring. This study advances the understanding of the effects of pre- and postnatal stressors in amygdala gene networks. The amygdala transcriptome was profiled on female and male pigs that were either exposed to viral-elicited MIA or not and were weaned or nursed. Overall, 111 genes presented interacting or independent effects of weaning, MIA, or sex (FDR-adjusted *P*-value <0.05). PIGY upstream reading frame and orthodenticle homeobox 2 are genes associated with MIA-related neurological disorders, and presented significant under-expression in weaned relative to nursed pigs exposed to MIA, with a moderate pattern observed in non-MIA pigs. Enriched among the genes presenting highly over- or under-expression profiles were 24 Kyoto Encyclopedia of Genes and Genomes pathways including inflammation, and neurological disorders. Our results indicate that MIA and sex can modulate the effect of weaning stress on the molecular mechanisms in the developing brain. Our findings can help identify molecular targets to ameliorate the effects of pre- and postnatal stressors on behaviors regulated by the amygdala such as aggression and feeding.

## Introduction

Weaning encompasses social, physical, and environmental stresses (Curley *et al.* 2009; Campbell *et al.* 2013). The separation of the offspring from their mother and familiar littermates, the physical handling and transport, comingling of unfamiliar individuals, new surroundings, and new feeding strategies can all contribute to the stress experienced by weaned rodents and pigs (Curley *et al.* 2009; Campbell *et al.* 2013). This event is accompanied by hormonal and immunological changes (Pie *et al.* 2004; Gimsa *et al.* 2018). Marked increases in plasma lymphocytic trapping and cortisol level were detected in pigs one day after weaning (Kick *et al.* 2012). Decreases in the concentrations of T cells, B cells, and natural killer cells in response to a stressful event corresponding with lymphocytic trapping were also observed in mice (Kick *et al.* 2012). A review of stress effects on pigs (Gimsa *et al.* 2018) highlighted the association of weaning with lower glucocorticoid receptor (GR) binding in the hippocampus and amygdala of 1-month-old pigs (Kanitz *et al.* 1998). Weaning together with isolation was associated with lower levels of the hydroxysteroid dehydrogenase enzyme that processes glucocorticoids, GR, and mineralocorticoid receptor (MR) in the prefrontal cortex of 2- to 3-week-old pigs (Poletto *et al.* 2006).

Infectious agents and environmental stressors can result in maternal immune activation (MIA) during gestation, and the ensuing changes in cytokine signaling can alter brain and nervous system development (Odorizzi and Feeney 2016; Prins *et al.* 2018). Using a viral model of MIA, we demonstrated the dysregulation of multiple genes and processes in the amygdalae of 3-week-old pigs, including immune response, neuroactive ligand–receptor, and glutamatergic pathways (Keever *et al.* 2020a, 2020b). This brain structure plays a central role in behaviors, including some that are sexually dimorphic or social in nature, and learning; thus, the plastic nature of this structure and its response to insults in crucial. Additionally, the amygdala regulates responses to pathogen infection and environmental stressors (Tian *et al.* 2015).

The response to postnatal stressors can be affected by prenatal MIA. Studies of MIA-associated behavior disorders such as schizophrenia spectrum disorders (SSD), autism spectrum disorders (ASD) (Knuesel *et al.* 2014; Canetta *et al.* 2016; Mattei *et al.* 2017) using rodent and primate models offer evidence of the combined effects of MIA and a second immune challenge (Maynard *et al.* 2001; Imanaka *et al.* 2006; Walker *et al.* 2009; Giovanoli *et al.* 2013). The double-hit hypothesis proposes that exposure to MIA elicits long-term neural and immune disruptions that subsequently modify the offspring’s response to a second immune challenge later in life (Bayer *et al.* 1999).

Offspring exposed to MIA can be more sensitive or more tolerant to a second stressor, and this response can modulate the incidence of behavioral disorders (Wang *et al.* 2007). Pigs exposed to MIA elicited by porcine reproductive and respiratory syndrome virus (PRRSV) during gestation and subsequently exposed to bacterial endotoxin lipopolysaccharide (LPS) at 2 weeks of age had higher circulating levels of proinflammatory cytokines and cortisol than pigs from PRRSV-infected gilts that did not receive the second challenge (Antonson *et al.* 2017).

Previous studies have reported molecular changes in the pig brain associated with weaning stress in the absence of MIA (Kanitz *et al.* 1998). Conversely, we focused on the study of MIA in absence of a second stressor and reported on the dysregulation of genes annotated to immune response pathways in the amygdalae of 3-week-old pigs exposed to MIA in the absence of weaning stress (Keever *et al.* 2020a). A study of the simultaneous effects of MIA and weaning stress on the amygdala pathways is necessary to assess possible interactions among both challenges.

The principal goal of this study is to advance the understanding of the effect of the double-hit paradigm on the amygdala gene networks of pigs. The combined effects on the molecular profiles of: (1) MIA stress elicited by PRRSV, (2) weaning stress, and (3) the interactions of these effects with sex were tested. A supporting objective is to reconstruct gene and transcription factor networks to augment the understanding of the effects of MIA and weaning stress on the interaction among genes and on gene regulatory elements. Molecular profiles of weaned pigs not exposed to MIA and of weaned pigs exposed to MIA were compared to existing profiles from pigs that did not experience weaning stress (Keever *et al.* 2020a). Conclusions drawn from these complementary approaches will aid in revealing the consequences of multiple stressors on the amygdala and may help in developing therapeutic strategies to mitigate these consequences.

## Materials and methods

### Animal experiments

Published protocols were used for all experimental procedures (Antonson *et al.* 2017, 2018; Keever *et al.* 2020a). Illinois Institutional Animal Care and Use Committee (IACUC) at the University of Illinois approved the animal studies, and they are in compliance with the USDA Animal Welfare Act and the NIH Public Health Service Policy on the Humane Care and Use of Animals.

Similar to our focused study of the effect of MIA alone (Keever *et al.* 2020a), PIC 359 boar semen was used to inseminate 205-day-old, PRRSV-negative Camborough gilts, which were born and raised at the University of Illinois at Urbana-Champaign (Antonson *et al.* 2017, 2018). At gestation day 69, the gilts were moved into disease containment chambers maintained at 22°C and a 12-h light/dark cycle with lights on at 7:00am, and they were fed daily 2.3 kg of a gestational diet with *ad libitum* water access. Following an acclimation period of 1 week, six gilts were inoculated intranasally with live PRRSV strain P129-BV (School of Veterinary Medicine at Purdue University, West Lafayette, IN, USA) using 5 mL of 1 × 10^5^ median tissue culture infectious dose (TCID_50_) diluted in sterile Dulbecco’s modified Eagle medium (DMEM; 5 mL total volume), while five gilts in the Control group were intranasally inoculated with a 5 mL of sterile DMEM. The timing of the intranasal inoculation corresponded to the last third of gestation in both pigs and humans, which coincides with the initiation of rapid fetal brain growth (Antonson *et al.* 2017, 2018; Keever *et al.* 2020a). Following inoculation, the PRRSV-challenged and control gilts were housed in separate containment chambers. PRRSV infection among PRRSV inoculated gilts and absence of infection among DMEM inoculated gilts was confirmed 1 week after exposure with a PCR test. The significant increase in body temperature and decrease in feed intake of PRRSV-challenged relative to control gilts resolved within 14 days postinjection.

Given that the average gestation lasting 114 days, an intramuscular injection of 10 mg of Lutalyse (dinoprost tromethamine, Pfizer, New York, NY, USA) was used to induce farrowing on gestation day 113 (Antonson *et al.* 2017, 2018), with gilts contained in individual farrowing crates of standard dimensions (1.83×1.83 m). Following farrowing, the gilts were fed 5 kg of a nutritionally complete diet twice daily for the lactating period with water available *ad libitum*. Intramuscular injections of iron dextran (100 mg/pig, Butler Schein Animal Health, Dublin, OH, USA) and Excede for Swine (25 mg/pig; Zoetis, Parsippany, NJ, USA) to control for respiratory diseases were administered to pigs. The pigs remained with the dam in the farrowing crates until weaning until 21 days of age, when approximately half of the pigs in each litter were weaned and the rest remained with the sow.

Weaned pigs were housed in groups of four, with *ad libitum* access to water and received a nutritionally complete diet for growing pigs. The nursing pigs that remained with the sow continued receiving the same feeding as before. The experiment concluded one day after weaning, a time point of high weaning stress. Reports confirm that pigs present heightened dysregulation of physiological stress indicators (e. g. blood neutrophil/lymphocyte ratio, glucose level, cortisol) 1 day after weaning (Puppe *et al.* 1997; Turpin *et al.* 2017), yet no significant effect on body weight (Puppe *et al.* 1997; Giroux *et al.* 2000a, 2000b; Turpin *et al.* 2017). Our analysis of body weight using a mixed-effect model include the effects of weaning, MIA, sex, and interactions and random effects of gilt and replicate confirmed the absence of significant stressor effects. The experimental design encompassed 48 pigs distributed between all eight groups representing MIA, weaning stress, and sex classes. To study the effect of MIA, we compared pigs that experienced maternal PRRSV-elicited activation (MIA group of pigs) relative to non-pigs from control gilts (non-MIA group of pigs). To study the effect of postnatal stress we compared pigs that were weaned at day 21 of age (weaned group of pigs) relative to pigs that remained with the gilt nursing until day 22 of age (nursed group of pigs). The comparison of male and female pigs enabled the study of sex effects.

### RNA extraction and sequencing

Pigs were anesthetized intramuscularly using a drug cocktail of telazol:ketamine:xylazine (50 mg of tiletamine; 50 mg of zolazepam) reconstituted with 2.5 mL ketamine (100 g/L) and 2.5 mL xylazine (100 g/L; Fort Dodge Animal Health, Fort Dodge, IA, USA) at a dose of 0.03 mL/kg body weight, following protocols (Antonson *et al.* 2017) at 22 days of age. An intracardiac injection of sodium pentobarbital (86 mg/kg body weight, Fata Plus, Vortech Pharmaceuticals, Dearborn, MI, USA) was used to euthanize the pigs after anesthetization. After euthanasia, the pig brains were removed, and the stereotaxic atlas of the pig brain (Félix *et al.* 1999) was used to identify the amygdalae. Amygdalae were dissected out, flash frozen on dry ice, and stored at −80°C following published protocols (Antonson *et al.* 2019). EZNA isolation kit (Omega Biotek, Norcross, GA, USA) was used to isolate RNA per manufacturer’s instructions. The RNA integrity numbers of the samples were above 7.3, indicating low RNA degradation. TruSeq Stranded mRNAseq Sample Prep kit’ (Illumina Inc, San Diego, CA, USA) was used to prepare RNAseq libraries, and libraries were quantitated by qPCR and sequenced on one lane on a NovaSeq 6000 for 151 cycles from each end of the fragments using NovaSeq S4 reagent kit. The bcl2fastq v2.20 conversion software was used to produce and demultiplex FASTQ files.

### RNA sequence mapping and differential expression analysis

Assessment of read quality found a minimum Phred score of 35 across all read positions using FASTQC (Andrews 2010), and, therefore, no reads were trimmed based on quality. Paired-end reads were aligned to the *Sus scrofa* transcriptome (version Sscrofa 11.1; Pruitt *et al.* 2007) and quantified using kallisto v. 0.43.0 (Bray *et al.* 2016) with default settings. The normalized (trimmed mean of *M*-values) gene expression values were described using a generalized linear model encompassing the effects of MIA group (MIA and non-MIA levels), weaning stress (weaned or nursed levels), sex (female or male levels), weaning stress-by-sex interaction, MIA-by-weaning stress interaction, and MIA-by-sex interaction. Few genes presented three-way interaction effects and, therefore, only two-way interactions and main effects were further considered. Genes supported by >5 transcripts per million (TPM) RNA molecules by each weaning-MIA-sex combination were analyzed for differential gene expression using edgeR (v. 3.14.0) in the R v. 3.3.1 environment (Robinson *et al.* 2010). A conservative cut-off for differential expression of FDR-adjusted *P*-value 5.0E-05 and log_2_(fold change between pig groups) >2 was considered to highlight strong trends. A positive log_2_(fold change) denotes over-expression in the first pig group relative to the second pig group in the comparison. A negative log_2_(fold change) denotes under-expression in the first pig group relative to the second pig group in the comparison. An extended list of genes at FDR-adjusted *P*-value <0.1 is included as Supplementary material. The raw and normalized gene expression levels for the 16,523 genes in the 48 samples are available in the National Center for Biotechnology Information (NCBI) Gene Expression Omnibus (GEO) database, series identifier GSE165059.

### Functional enrichment, network inference, and transcription factor analysis

Identification of the over-represented Kyoto Encyclopedia of Genes and Genomes (KEGG) pathways (Kanehisa and Goto 2000; Kanehisa 2019) among all genes analyzed for the interaction and main effects of weaning stress, MIA, and sex was performed using the Gene Set Enrichment Analysis (GSEA) approach with the WebGESTALT software (Liao *et al.* 2019). The complete list The GSEA used the information from all genes analyzed, ranked from the most over-expressed to the most under-expressed to identify over-represented pathways within both profiles. The normalized enrichment score (NES) of the pathways was calculated using the maximum deviation of the cumulative sum based on the signed fold-change of the ranked genes divided by the average of the permutated enrichment scores. For the enrichment analysis, *Sus scrofa* was selected as the organism of interest, and a minimum of five genes and a maximum of 2000 genes per category were used. Statistical significance was determined by calculating the FDR-adjusted *P*-value from 1000 permutations. A conservative cut-off for differential expression of FDR-adjusted *P*-value 5.0E-05 and NES >2 was considered to highlight highly enriched pathways.

The sign of the NES from GSEA offered insights into the predominance of the gene expression profile within the enriched categories for each effect tested. Pathways characterized with an NES >0 are enriched among the genes over-expressed while the pathways characterized with an NES <0 are enriched among the genes under-expressed. The consideration of the estimable functions and comparison against the pair-wise contrast of gene expression levels between pig groups enabled the characterization of the predominant profiles within positive (NES >0) and negative (NES <0) group. For the weaning-by-MIA interaction effect, NES >0 denoted gene over-expression in non-MIA nursed relative to MIA weaned pigs and NES <0 denoted gene under-expression in non-MIA nursed relative to MIA weaned pigs. For the weaning-by-sex interaction effect, NES >0 denoted gene over-expression in nursed males relative to weaned females, and NES <0 denoted gene under-expression in nursed males relative to weaned females. For the MIA-by-sex interaction effect, NES >0 denoted gene over-expression in MIA females relative to MIA males, and NES <0 denoted gene under-expression in MIA females relative to MIA males. For the weaning effect, NES >0 denoted over-expression in weaned relative to nursed pigs, and NES <0 denoted over-expression in nursed relative to weaned pigs. For the MIA effect, NES >0 denoted gene over-expression in non-MIA relative to MIA pigs, and NES <0 denoted gene over-expression in MIA relative to non-MIA pigs. For the sex effect, NES >0 denoted gene over-expression in males relative of females, and NES <0 gene denoted over-expression in females relative of males. Positive-fold change and NES values from GSEA for the main effects of MIA, weaning stress, and sex indicated over-expression in weaned relative to nursed pigs, non-MIA relative to MIA pigs, and male relative of female pigs.

The genes that showed significant MIA-by-weaning stress effect and differential expression between nursed and weaned pigs in the MIA and non-MIA groups (FDR-adjusted *P*-value <0.1) were further studied. First, the genes presenting weaning effects within MIA group were used to build the framework of gene networks that could help uncover distinct gene interaction patterns between weaning groups. A preliminary identification of interacting proteins coded by the differentially expressed genes in our study was generated using STRING v. 11.0 (Szklarczyk *et al.* 2019). The relationships between genes depicted in STRING are based on known data contained in manually curated databases, such as BioCyc (Karp *et al.* 2019), Gene Ontology (Ashburner *et al.* 2000; Carbon *et al.* 2019), and KEGG (Kanehisa and Goto 2000; Kanehisa 2019), and predicted protein–protein interactions mined from high throughput experiments and primary literature. To understand the potential changes in the relationship between genes elicited by MIA in either weaning group, the STRING networks were subsequently enhanced in Cytoscape v. 3.8.1 (Shannon *et al.* 2003) by including the gene expression profiles measured in the present study. In the reconstructed networks, the color of the gene reflects the sign of the fold change where red denotes over-expression in weaned relative to nursed pigs and blue denotes the opposite pattern,

The set of genes presenting MIA interaction effect was also studied with the goal of identifying shared transcription regulators. The identification of potential transcription factors and motifs over-represented among the target genes was carried out using the iRegulon application (Janky *et al.* 2014) in Cytoscape v. 3.8.1 (Shannon *et al.* 2003). Default settings of receiver operator curve estimation of the area under the curve threshold equal to 0.03 and motif similarity of FDR-adjusted *P*-value <0.001 were used to rank motifs based on position weight matrices, and transcription factors capable of regulating differentially expressed target genes were deemed enriched when supported by >15% of the input genes and NES ≥3 (Janky *et al.* 2014).

### Data availability

The raw and normalized gene expression levels are available in the National Center for Biotechnology Information (NCBI) Gene Expression Omnibus (GEO) database, series identifier GSE165059. Supplementary material is available at figshare DOI: https://doi.org/10.25387/g3.13607600.

## Results

### Maternal immune activation and sequencing metrics

The sequencing produced 6.59E + 09 reads across all 48 libraries. The median number of reads per pig group is 1.35E + 08 for weaned pigs, 1.22E + 08 for nursed pigs, 1.35E + 08 for non-MIA pigs, 1.31E + 08 for MIA pigs, 1.34E + 08 for females, and 1.35E + 08 for males. The median number of reads mapped by the kallisto software per group is 6.77E + 07 for weaned pigs, 6.62E + 07 for nursed pigs, 6.81E + 07 for non-MIA pigs, 6.54E + 07 for MIA pigs, 7.03E + 07 for females, and 6.54E + 07 for males. The analysis of 16,529 genes that had >5 mapped read counts per pig group enabled testing the factors of weaning, MIA, sex, and interaction on the gene expression profiles.

### Effects of weaning stress, MIA, and sex on the amygdala pathways

Table 1 presents a summary of the KEGG pathways enriched in at least one tested interaction or main effect at FDR-adjusted *P*-value <0.05 and NES>|2|. Supplementary Table S1 presents an extended list of the 115 KEGG pathways enriched in at least one effect at *P*-value <0.05. Weaning stress showed significant effects, interacting with other factors or alone, on the gene expression profiles in the amygdala of pigs. The GSEA of the genes ranked from highly over-expressed to highly under-expressed identified 21 KEGG pathways enriched (Table 1) among the genes presenting MIA-by-weaning interaction effect, and 13 KEGG pathways enriched among genes presenting weaning stress effect. An extended list of pathways at *P*-value <0.05 is included in Supplementary Table S1.

**Table 1:** Pathways enriched in at least one effect including interaction or main effect of weaning (Wea), maternal immune activation (MIA), and sex at *P*-value <5.0E-05, and normalized enrichment score |NES|>2

KEGG pathway	Wea×MIA		Wea×Sex		Wea		MIA×Sex			MIA		Sex	
N	*P*	N	*P*	N	*P*	N		*P*	N	*P*	N	*P*

ssc04080: Neuroactive ligand–receptor	−1.5	6.E-03	−2.0	1.E-05	1.5	1.E-02	−2.0		1.E-05	−1.4	5.E-02		
ssc05010: Alzheimer disease	−1.3	5.E-02	−1.9	1.E-03			−2.0		1.E-05				
ssc04514: Cell adhesion molecules	1.9	1.E-05			−1.5	6.E-03	1.4		3.E-02	−1.8	2.E-03		
ssc04932: Non-alcoholic fatty liver disease	−1.6	6.E-03	−2.0	1.E-05			−2.1		1.E-05	−1.8	5.E-03	1.5	6.E-02
ssc05012: Parkinson disease	−1.5	4.E-02	−2.0	1.E-05			−2.1		1.E-05	−1.4	4.E-02	1.6	5.E-02
ssc04659: Th17 cell differentiation	2.0	1.E-05			−1.5	1.E-02	1.7		8.E-03	−1.5	4.E-02		
ssc04623: Cytosolic DNA-sensing pathway	2.1	1.E-05			−1.6	2.E-02	1.4		7.E-02				
ssc04145: Phagosome	2.2	1.E-05			−1.4	2.E-02	1.6		1.E-05	−1.9	1.E-03		
ssc05164: Influenza A	2.1	1.E-05			−1.7	1.E-05	2.0		1.E-05	−1.7	3.E-03		
ssc04658: Th1 and Th2 cell differentiation	2.1	1.E-05			−1.6	1.E-02	1.7		1.E-05	−1.5	4.E-02		
ssc05169: Epstein–Barr virus infection	2.1	1.E-05			−1.8	1.E-05	1.6		1.E-05	−1.9	1.E-05		
ssc05323: Rheumatoid arthritis	2.2	1.E-05					1.6		1.E-02	−1.6	1.E-02		
ssc05168: Herpes simplex infection	2.2	1.E-05			−1.7	2.E-03	2.0		1.E-05	−1.7	9.E-03		
ssc00190: Oxidative phosphorylation	−1.3	9.E-02	−1.9	3.E-03			−2.3		1.E-05	−1.3	7.E-02	1.5	7.E-02
ssc03010: Ribosome			−2.2	1.E-05	2.2	1.E-05	−2.5		1.E-05	−1.9	2.E-03	1.4	1.E-01
ssc04612: Antigen processing, presentation	2.6	1.E-05			−2.2	1.E-05	2.5		1.E-05	−1.6	2.E-02	−1.7	6.E-03
ssc04940: Type I diabetes mellitus	2.4	1.E-05			−2.1	1.E-05	2.4		1.E-05	−1.7	1.E-02	−1.7	5.E-03
ssc05320: Autoimmune thyroid disease	2.4	1.E-05	−1.5	5.E-02	−2.1	1.E-05	1.6		2.E-02	−1.9	2.E-03	1.4	1.E-01
ssc05322: Systemic lupus erythematosus	2.4	1.E-05			−1.7	6.E-03	1.9		1.E-05	−1.5	3.E-02		
ssc05332: Graft-*vs*-host disease	2.4	1.E-05			−2.2	1.E-05	2.3		1.E-05	−1.7	9.E-03	−1.7	2.E-02

Sex effect where NES >0 denotes gene over-expression in males relative of females, and NES <0 denotes gene over-expression in females relative of males.

N, NES; Wea×MIA, weaning-by-MIA interaction effect where NES >0 denotes gene over-expression in non-MIA nursed relative to MIA weaned pigs, and NES <0 denotes gene under-expression in non-MIA nursed relative to MIA weaned pigs; Wean×Sex, weaning-by-sex interaction effect where NES >0 denotes gene over-expression in nursed males relative to weaned females, and NES <0 denotes gene under-expression in nursed males relative to weaned females; MIA×Sex, MIA-by-sex interaction effect where NES >0 denotes gene over-expression in MIA females relative to MIA males, and NES <0 denotes gene under-expression in MIA females relative to MIA males; Wea, weaning effect where NES >0 denotes gene over-expression in weaned relative to nursed pigs, and NES <0 denotes gene over-expression in nursed relative to weaned pigs; MIA, maternal immune activation effect where NES >0 denotes gene over-expression in non-MIA relative to MIA pigs, and NES denotes gene over-expression in MIA relative to non-MIA pigs; and

*P*, *P*-value <5.0E-05 corresponds to a false discovery rate *P*-value <5.0E-02.

Among the enriched KEGG pathways in Table 1, there is a predominance of immune- and neurologically related functional categories. Based on the NES values in Table 1, there is a prevalence of GSEA enrichment among genes under-expressed in weaned relative to nursed pigs, under-expressed in MIA relative to non-MIA pigs. The more significant enrichments of KEGG pathways were identified among genes presenting weaning-by-MIA, MIA-by-sex, weaning, and MIA effects.

### Effects of weaning stress, MIA, and sex on the gene expression profiles in the amygdala

**Figure 1:**
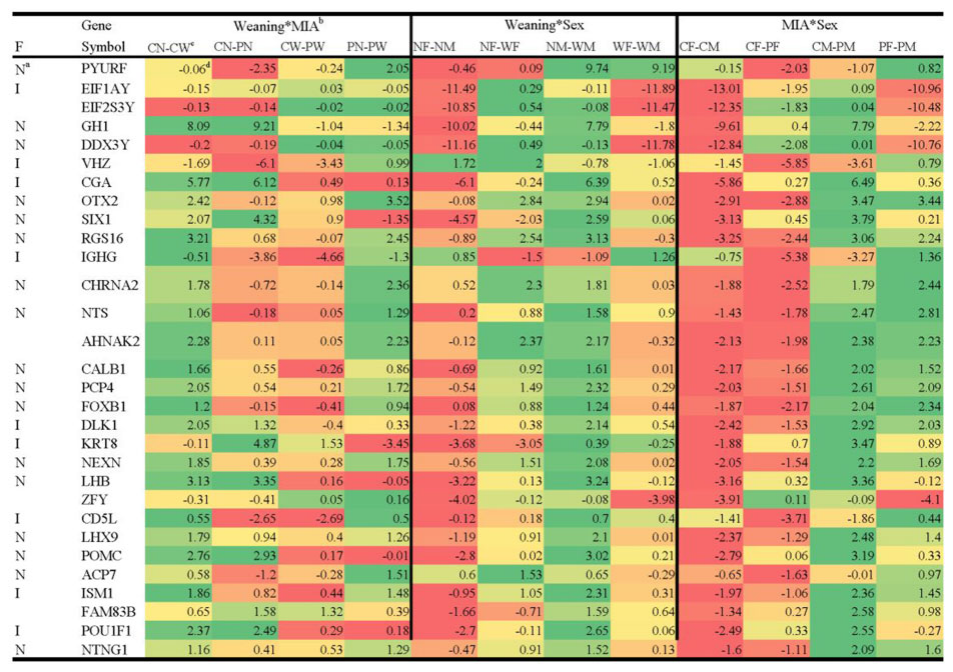
Heatmap listing the differential expression [log_2_(fold change between pig groups)] of genes that presented at least one significant interaction or main effect of weaning, maternal immune activation at *P*-value <5.0E-05 and |log_2_(fold change between pig groups)|>2.0. ^a^Functional annotation of the gene; N, neuro-associated or I, immune-associated function. ^b^Weaning×MIA: log_2_(fold change between pig groups) characterizing the weaning-by-maternal immune activation effect; Weaning×Sex: log_2_(fold change between pig groups) characterizing the weaning-by-sex effect; MIA×Sex: log_2_(fold change between pig groups) characterizing the maternal immune activation-by-sex effect. ^c^Log_2_(fold change of pig group X relative to group Y)=log_2_(X/Y)=log_2_(X) – log_2_(Y). The pig groups compared included: non-MIA or control nursed pigs (CN), non-MIA weaned pigs (CW), PRRSV-elicited MIA nursed pigs (PN), MIA weaned pigs (PW), nursed females (NF), nursed males (NM), weaned females (WF), weaned males (PM), non-MIA females (CF), non-MIA males (CM), PRRSV-elicited MIA females (PF), MIA males (PM). ^d^Red (green) denotes under(over)-expression in the X or first pig group relative to the Y or second pig group.

Overall, 111 genes had significant (FDR-adjusted *P*-value <0.05) effects of weaning stress, MIA, and sex interacting or acting independently. At *P*-value <0.005, a total of 254 genes exhibited significant changes in the profile associated with the previous effects. Table 2 summarizes the genes that presented one or more significant main or interaction effects at |log_2_(fold change between pig groups)|>2 and *P*-value <5.0E-05 (approximately FDR-adjusted *P*-value <0.05), and Figure 1 provides the corresponding expression profiles characterized by the log_2_(fold change between pig groups). Supplementary Table S2 includes an extended list of 71 genes that presented at least one significant effect at FDR-adjusted *P*-value <0.1.

**Table 2:** *P*-value (*P*) and false discovery rate-adjusted *P*-value (FDR) of genes that presented at least one significant interaction or main effect of weaning, maternal immune activation at *P*-value <5.0E-05 and |log_2_(fold change between pig groups)|>2.0

Gene	MIA	MIA	MIA×S	MIA×S	W×MIA	W×MIA	Sex	Sex	W×Sex	W×Sex	Wea	Wea
Symbol	*P*	FDR	*P*	FDR	*P*	FDR	*P*	FDR	*P*	FDR	*P*	FDR
PYURF	4.E-25	7.E-21	4.E-15	2.E-11	2.E-17	2.E-13	2.E-20	3.E-17	2.E-36	4.E-32	2.E-20	4.E-16
EIF1AY	9.E-03	1.E + 00	1.E-02	1.E + 00	1.E-01	1.E + 00	6.E-34	5.E-30	2.E-01	1.E + 00	2.E-01	1.E + 00
EIF2S3Y	6.E-02	1.E + 00	9.E-02	1.E + 00	8.E-01	1.E + 00	2.E-32	8.E-29	5.E-01	1.E + 00	4.E-01	1.E + 00
GH1	8.E-01	1.E + 00	2.E-19	3.E-15	8.E-01	1.E + 00	2.E-32	8.E-29	7.E-22	5.E-18	4.E-01	1.E + 00
DDX3Y	1.E-01	1.E + 00	1.E-01	1.E + 00	4.E-01	1.E + 00	9.E-32	3.E-28	3.E-01	1.E + 00	3.E-01	1.E + 00
VHZ	8.E-20	7.E-16	1.E-05	8.E-03	2.E-06	5.E-03	8.E-04	3.E-01	2.E-02	1.E + 00	4.E-04	9.E-01
CGA	9.E-01	1.E + 00	2.E-13	5.E-10	5.E-01	1.E + 00	3.E-19	4.E-16	1.E-14	8.E-11	4.E-01	1.E + 00
OTX2	9.E-10	4.E-06	6.E-17	3.E-13	3.E-08	1.E-04	2.E-09	2.E-06	5.E-06	1.E-02	2.E-01	1.E + 00
SIX1	5.E-01	1.E + 00	3.E-08	3.E-05	2.E-01	1.E + 00	1.E-13	1.E-10	9.E-09	4.E-05	2.E-04	5.E-01
RGS16	7.E-07	1.E-03	1.E-13	4.E-10	1.E-04	2.E-01	3.E-09	3.E-06	1.E-05	2.E-02	9.E-01	1.E + 00
IGHG	9.E-11	5.E-07	5.E-02	1.E + 00	3.E-01	1.E + 00	3.E-01	1.E + 00	9.E-01	1.E + 00	5.E-01	1.E + 00
CHRNA2	2.E-07	4.E-04	1.E-10	3.E-07	1.E-04	2.E-01	3.E-05	1.E-02	1.E-03	1.E + 00	7.E-01	1.E + 00
NTS	4.E-05	4.E-02	1.E-09	2.E-06	2.E-02	1.E + 00	2.E-04	8.E-02	5.E-03	1.E + 00	3.E-01	1.E + 00
AHNAK2	8.E-05	7.E-02	1.E-09	2.E-06	8.E-03	1.E + 00	5.E-05	3.E-02	2.E-02	1.E + 00	2.E-01	1.E + 00
CALB1	1.E-04	8.E-02	1.E-09	2.E-06	2.E-02	1.E + 00	2.E-06	1.E-03	1.E-03	8.E-01	4.E-01	1.E + 00
PCP4	4.E-04	2.E-01	2.E-09	2.E-06	8.E-03	1.E + 00	1.E-05	7.E-03	1.E-03	8.E-01	1.E + 00	1.E + 00
FOXB1	2.E-05	2.E-02	2.E-09	2.E-06	7.E-02	1.E + 00	6.E-05	3.E-02	2.E-02	1.E + 00	6.E-01	1.E + 00
DLK1	6.E-03	1.E + 00	2.E-09	3.E-06	5.E-01	1.E + 00	2.E-06	2.E-03	3.E-03	1.E + 00	1.E + 00	1.E + 00
KRT8	1.E-02	1.E + 00	7.E-05	3.E-02	3.E-01	1.E + 00	3.E-09	3.E-06	2.E-04	2.E-01	1.E-05	5.E-02
NEXN	1.E-04	9.E-02	7.E-09	8.E-06	1.E-03	1.E + 00	5.E-06	3.E-03	6.E-04	6.E-01	6.E-01	1.E + 00
LHB	3.E-01	1.E + 00	1.E-04	6.E-02	5.E-01	1.E + 00	1.E-08	1.E-05	8.E-05	1.E-01	6.E-01	1.E + 00
ZFY	1.E + 00	1.E + 00	8.E-01	1.E + 00	8.E-01	1.E + 00	2.E-08	1.E-05	1.E + 00	1.E + 00	7.E-01	1.E + 00
CD5L	3.E-08	8.E-05	1.E-02	1.E + 00	4.E-01	1.E + 00	4.E-03	1.E + 00	2.E-01	1.E + 00	6.E-01	1.E + 00
LHX9	8.E-04	5.E-01	3.E-08	3.E-05	1.E-02	1.E + 00	6.E-07	4.E-04	1.E-03	8.E-01	5.E-01	1.E + 00
POMC	9.E-01	1.E + 00	7.E-06	4.E-03	8.E-01	1.E + 00	4.E-08	3.E-05	2.E-05	3.E-02	8.E-01	1.E + 00
ACP7	2.E-07	4.E-04	5.E-07	4.E-04	4.E-08	1.E-04	3.E-04	1.E-01	1.E-05	2.E-02	1.E-02	1.E + 00
ISM1	2.E-03	1.E + 00	2.E-07	1.E-04	8.E-03	1.E + 00	7.E-06	4.E-03	4.E-04	4.E-01	6.E-01	1.E + 00
FAM83B	2.E-02	1.E + 00	2.E-07	2.E-04	9.E-04	1.E + 00	6.E-07	5.E-04	8.E-06	2.E-02	1.E-03	1.E + 00
POU1F1	8.E-01	1.E + 00	8.E-04	3.E-01	7.E-01	1.E + 00	2.E-07	2.E-04	5.E-05	7.E-02	6.E-01	1.E + 00
NTNG1	2.E-03	8.E-01	4.E-07	3.E-04	1.E-02	1.E + 00	1.E-04	5.E-02	7.E-03	1.E + 00	5.E-01	1.E + 00

MIA, maternal immune activation effect; MIA×S, maternal immune activation-by-sex interaction effect; W×MIA, weaning-by-maternal immune activation interaction effect; sex, sex effect; W×Sex, weaning-by-sex interaction effect; Wea, weaning effect.

*P*-value <5.0E-05 corresponds to approximate false discovery rate-adjusted *P*-value <5.0E-02.

Overall, 54 genes presented significant (FDR-adjusted *P*-value <0.05) interaction effects and 68 genes presented significant main effects. The majority of the genes presenting significant main effect included a significant interaction of this effect with the remaining effects (Table 2 and Figure 1, Supplementary Table S2). Among the 71 genes that presented one or more significant effects at FDR-adjusted *P*-value <0.1 (Supplementary Table S2), 87% were over-expressed in non-MIA pigs, 86% were over-expressed in males, and 70% were over-expressed in weaned pigs, relative to the alternative factor levels.

Among the genes that presented at least one significant effect at FDR-adjusted *P*-value <0.05 and |log_2_(fold change)|>2, (Figure 1), the majority were under-expressed in female relative to male nursed pigs under-expressed in female relative to male non-MIA pigs, over-expressed in nursed relative to weaned males, and over-expressed in non-MIA relative to MIA males.

### Effects of weaning stress and MIA on gene networks and regulatory mechanisms in the amygdala

The simultaneous study of the profile and interconnection between the genes that presented MIA-by-weaning stress interaction offered insights into the potential impact of the first and second stressors on the relationship between the genes.

**Figure 2:**
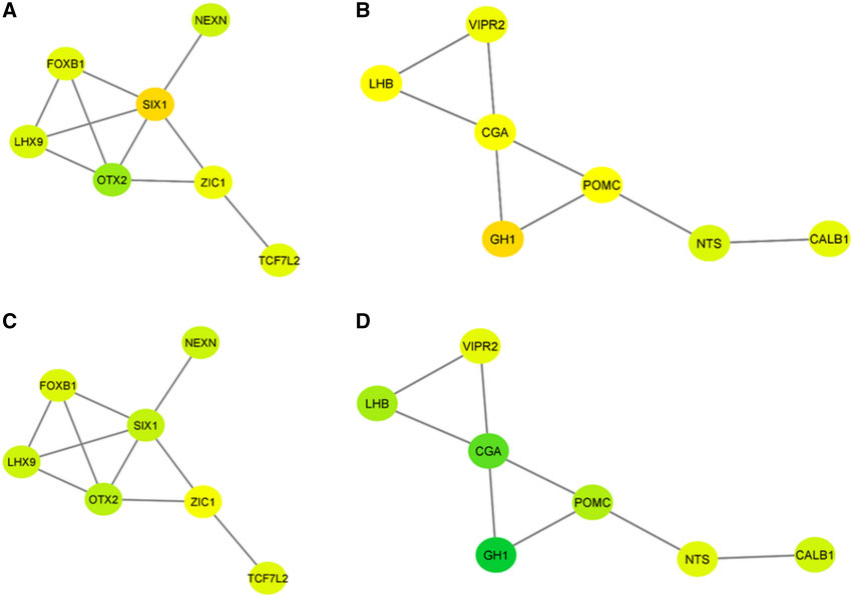
Gene network submodules of the gene orthodenticle homeobox 2 (A, C) and the gene vasoactive intestinal peptide receptor 2 (B, D). Modules (A) and (B) depict under- (blue) and over- (red) expression between MIA weaned and nursed pigs while modules (C) and (D) depict under- (blue) and over- (red) expression between non-MIA weaned and nursed pigs.

Figures 2, A and B depict two modules of genes that presented a MIA-by-weaning interaction effect where the blue and red denote genes under- and over-expressed in weaned relative to nursed pigs exposed to MIA. Figures 2, C and D depict two modules of genes that presented a MIA-by-weaning interaction effect where the blue and red denote genes under- and over-expressed in non-MIA weaned relative to nursed pigs. Higher color intensities denote higher fold changes that are reported in Table 2 and Figure 1 and Supplementary Table S2. The depicted genes included nexilin F-actin binding protein (NEXN), forhead box B1 (FOXB1), LIM homeobox 9 (LHX9), orthodenticle homeobox 2 (OTX2), SIX homeobox 1 (SIX1), Zic family member 1 (ZIC1), transcription factor 7 like 2 (TCF7L2), vasoactive intestinal peptide receptor 2 (VIPR2), luteinizing hormone subunit beta (LHB), glycoprotein hormones, alpha polypeptide (CGA), growth hormone 1 (GH1), proopiomelanocortin (POMC), neurotensin (NTS), and calbindin 1 (CALB1).

The comparison of expression patterns in consideration of the relationships between gene nodes highlights the profile change between MIA (Figure 2A) and non-MIA (Figure 2C) pigs in response to weaning stress in the module of the interacting OTX2, ZIC1, and TCFL2. The changes between MIA groups in the neuropeptide hormone gene module are the attenuation in the weaning effect on the profiles of VIPR2, POMC, and LHB that MIA exerted. This attenuation is visualized by the lower color intensity of the nodes in Figure 2B relative to Figure 2D.

The study of the enrichment of transcription factors among the genes that presented MIA-by-weaning stress interaction advanced the understanding of the impact of the first and second stressors on the co-expression profile of these genes. Transcription factors RAD21 and SUZ12 were enriched. Among the potential targets of RAD21 are three genes that shared the same differential expression between weaned and nursed in the MIA (Figures 2, A and B) and non-MIA groups (Figures 2, C and D). RAD21 targets POMC andGH1 and the differential expression of these genes between nursed and weaned pigs was consistent across MIA groups. RAD21 was over-expressed in weaned relative to nursed pigs and over-expressed in MIA relative to non-MIA groups.

The detected enrichment of the transcription factor SUZ12 among genes presenting MIA-by-weaning effect can be related to the over-expression of this gene in the MIA relative to non-MIA group. Targets of SUZ12 include FOXB1, a gene that was over-expressed in MIA pigs relative to non-MIA pigs. OTX2 and ZIC1 are also targets of SUZ12 and presented different profiles in response to weaning across MIA groups.

## Discussion

Studies in rodents and humans surmise that prenatal immune challenge can augment (*i.e.*, sensitization) or reduce (*i.e.*, tolerance) the vulnerability of the brain and ensuing behavior disruption to subsequent challenges (Eklind *et al.* 2001, 2005; Wang *et al.* 2007). Our results offer evidence that the double-hit hypothesis postulated in studies of behavior disorders associated with amygdala functions, including ASD and SSD in humans (Feigenson *et al.* 2014) and comparable behaviors in rodents (Wang *et al.* 2007), is germane to molecular pathways in the amygdala of weaned pigs exposed to viral infection during gestation. Moreover, we uncovered sex-dependent effects of pre and postnatal stressors in pigs.

The present study evaluated the effects of MIA and the postnatal stress of weaning on the amygdala transcriptome of female and male pigs. The mapped reads averaged 67% per sample and pig group, and is consistent with the mapping by kallisto software in other pig RNA-seq studies (Tan *et al.* 2017). The experimental design enabled the simultaneous testing of the impact of MIA and weaning within sex, in addition to detecting the independent effects of each stressor. The GSEA of the genes analyzed yielded 24 KEGG pathways enriched in at least one tested effect at FDR-adjusted *P*-value <0.05 (Table 1) and the expression profiles of 118 genes presented MIA, weaning, sex, effects at FDR-adjusted *P*-value <0.5. Pathways associated with immune and neurological processes dominated the enriched categories (Tables 1 and 2).

### Effect of weaning-by-MIA interaction on amygdala pathways

The majority of the KEGG pathways enriched among the genes presenting weaning-by-MIA interaction effects were characterized by pigs in the MIA weaned group presenting decreased expression levels that were comparable to the non-MIA nursed group, relative to MIA nursed and non-MIA weaned groups. This blunting effect of the double hit indicates a prolonged effect of MIA dampening the immune response or related pathways, following the second stress of weaning. For certain processes, the decreased gene expression profiles in the MIA weaned pigs may sensitize the amygdala, making this brain structure more vulnerable to the second stressor. In contrast, for other processes, the decreased gene expression profiles in the MIA weaned pigs may afford tolerance, lowering the amygdala response to the postnatal stressor. This duality can be observed in molecular mechanisms associated with immune functions that include genes acting in feedforward and feedback manner, such as those detected in the present study.

The infection- and inflammation-associated pathways enriched among the genes presenting decreased expression in MIA weaned pigs included the Epstein–Barr virus infection (ssc05169) and influenza A (ssc05164) pathways (Table 1, Supplementary Table S1). Weaning can trigger inflammatory processes and rats that have undergone maternal separation presented over-expression of inflammation-related genes in the microglia within the dorsal striatum and nucleus accumbens, and sustained hippocampal inflammation (Banqueri *et al.* 2019). Also, the pro-inflammatory cytokine TNF-α was over-expressed in the hippocampi of piglets exposed to MIA via PRRSV (Antonson *et al.* 2018). These reports illustrate responses to a single stressor, whereas a growing number of studies report that a first stressor may confer resilience to future insults (Ashby *et al.* 2010; Yee *et al.* 2011a; Goh *et al.* 2020). Consistent with our findings a study of mice exposed to MIA triggered by polyinosinic:polycytidylic acid (Poly(I:C)) or social isolation reported higher cytokine expression in either condition whereas cytokine levels were lower in the brains of mice that experienced a double-hit, MIA, and social isolation (Yee *et al.* 2011b).

Enriched among the genes exhibiting a significant MIA-by-weaning interaction effect were several pathways associated with autoimmune diseases, such as autoimmune thyroid disease (ssc05320) and rheumatoid arthritis (ssc05323, Table 1). The detected pathways, characterized genes under-expressed in MIA weaned pigs, are consistent with reports of a link between dysregulation of autoimmune pathways and SSD-related behaviors (Eaton *et al.* 1992). In agreement with our results, autoimmune diseases associated with the thyroid have an increased prevalence among parents of patients with SSD compared to parents of control subjects (Eaton *et al.* 2006). Additionally, both LPS-induced MIA and early weaning have been shown to increase the susceptibility of mice and rats, respectively, to experimental autoimmune encephalomyelitis (Laban *et al.* 1995; Zager *et al.* 2015). Similarly, pathways associated with cells involved in the pathogenesis of autoimmune disease, including Th1 and Th2 cell differentiation (ssc04658) and Th17 cell differentiation (ssc04659), were also enriched among the genes presenting a significant MIA-by-weaning interaction and under-expressed in MIA weaned pigs.

The cell adhesion molecules (CAMs) pathway (ssc04514) was enriched among genes exhibiting MIA-by-weaning interaction effects (Table 1). The over-representation of the CAMs pathway among genes under-expressed in MIA weaned pigs is also supported by the enrichment of this pathway in genes under-expressed in the cortex of ASD patients, with similar results found in the brains of rats subject to LPS-induced MIA (Lombardo *et al.* 2018). Our characterization of the double hit impact offered evidence that MIA can disrupt the immune response to weaning stress.

### Effects of weaning and MIA on gene interactions and regulation

The reconstruction of gene networks in consideration of the differential expression patterns between nursed and weaned pigs and contrast between MIA and non-MIA groups advanced the recognition of the interplay between expression profiles in response to gestation and postnatal stressors. The comparison of networks underscored the diminished effect of weaning stress on the expression of neuropeptide hormone-related genes, including VIPR2, POMC, and LHB, among MIA relative to non-MIA pigs. The long-lasting impact of stressors during gestation on the POMC regulation and permanent effects on the hypothalamic–pituitary–adrenal axis function have been noted (Amath *et al.* 2012). Also, the expression level of LHB was altered in the peripheral blood of subjects with ASD (Kuwano *et al.* 2011), and polymorphisms and copy number variants in VIPR2 have been associated with ASD and SSD (Firouzabadi *et al.* 2017).

The weakened effect of weaning on the expression of multiple genes that have signaling roles suggests an antagonistic relationship between immune activation during gestation and weaning stress. This result aligns with the double-hit hypothesis (Maynard *et al.* 2001; Imanaka *et al.* 2006; Walker *et al.* 2009; Giovanoli *et al.* 2013) that gestational immune challenge can elicit long-term molecular disruptions that alter the offspring’s response to another stressor later in life.

The expression patterns in Figure 1 suggest that some targets of the transcription factor ZBTB4 are less affected by MIA (*e.g.*, SIX1), whereas targets that have a more moderate differential expression in response weaning are affected by MIA (*e.g.*, TCF7L2, ZIC1, FOXB1). The exception was OTX2 that was over-expressed in MIA pigs relative to non-MIA pigs. The epigenetic action of ZBTB4 has been associated with the MIA-related condition of ASD (Smith *et al.* 2010).

The profiles of the transcription factors RAD21 and SUZ12 were associated with multiple genes exhibiting MIA-by-weaning effect and were over-expressed in MIA relative to non-MIA pigs, albeit at lower statistical significance than the target genes. RAD21 was negatively associated with the expression of neuropeptide gene GH1 and positively associated with the expression of POMC and NTS. A key role of RAD21 in brain development has been proposed (Benitez-Burraco and Uriagereka 2016), and RAD21 has been associated with ASD (Persico *et al.* 2020). SUZ12 is considered a risk gene for ASD (Crawley *et al.* 2016) and could target FOXB1, OTX2, and ZIC1. The effect of SUZ12 is evident in the different impact of weaning across MIA groups on the target genes.

### Sex-dependent effects of weaning and MIA on amygdala pathways

Notably, the most enriched pathways among the genes that presented weaning-by-sex interaction effects were characterized by gene under-expression in weaned males and nursed female relative to weaned female and nursed male groups (Table 1). This pattern suggests that weaning stress depresses gene expression in males while heightening the level in females. Pathways involved in neurodegenerative diseases, including AD and Parkinson’s disease, were enriched among genes with a profile of over-expression in weaned females (Table 1**)**.

The stress triggered by maternal separation has been associated with long-term modifications in midbrain dopaminergic neurons, primarily in females (Chocyk *et al.* 2011). This pattern is consistent with the over-expression of genes such as OTX2 that modulates midbrain GABA, dopamine, and serotonin-releasing neurons in weaned females. The enrichment of the oxidative phosphorylation pathway (ssc00190, Table 1) is consistent with findings that short-term treatment of neurons with corticosterone, a mediator of the stress response, increases mitochondrial oxidation potential within neurons, exerting a neuroprotective effect against stress (Du *et al.* 2009).

The enrichment of the neuroactive ligand–receptor interaction pathway (ssc04080) among the genes under-expressed in weaned males and nursed females (Table 1**)** is consistent with a similar enrichment among genes dysregulated in the amygdalae of mice exposed to psychological and physical stress (Sun *et al.* 2019). The neuroactive ligand–receptor interaction pathway was enriched among genes over-expressed in the amygdalae of mice that are resilient to chronic unpredictable mild stress (Shen *et al.* 2019). The retrograde endocannabinoid signaling pathway (ssc04723, Supplementary Table S1) was also enriched among genes exhibiting a profile of under-expression in weaned males and nursed females. Increased endocannabinoid signaling is associated with a dampened stress response (Hillard 2014). The neuroactive ligand–receptor and the retrogragde endocannabinoid signaling pathways support the hypothesis that females could curb the effects of MIA on behavioral or neurological mechanisms regulated by the amygdala.

### Effects of weaning stress, MIA, and sex on genes profiles

Multiple genes presenting significant differential expression in the amygdala of pigs across weaning, MIA, and sex groups (Table 2 and Figure 1, Supplementary Table S2) have been associated with stress effects. Genes associated with neural pathways and neurological disorders and immune-related functions presented significant weaning-by-MIA interaction effect, including OTX2, cholinergic receptor nicotinic alpha 2 subunit (CHRNA2), acid phosphatase 7, immunoglobulin heavy variable 3-23 (VHZ), and a gene annotated to the ribosome pathway PIGY upstream reading frame (PYURF) (Tables 1 through 3 with Tables 1 and 2). For these genes, the double hit mode of action was characterized by reversal of the weaned effect between the non-MIA and MIA groups with the exception of MAGED4, which had a weakened weaning effect in MIA relative to non-MIA pigs.

The expression patterns of OTX2 in response to weaning and MIA effects is in agreement with reports of under-expression in the amygdala of rats exposed to neonatal stress and displaying anxiety as adults (Sarro *et al.* 2014). OTX2 modulates midbrain GABA, dopamine, and serotonin-releasing neurons and binding of OTX2 to perineuronal nets (biomarkers for SSD) grants neuroplasticity (Sarro *et al.* 2014). Postnatal stress was also associated with the under-expression of OTX2 in the ventral tegmental area in mice (Pena *et al.* 2017). MAGED4 has been linked to ASD (Kanduc and Polito 2018), and CHRNA2 was differentially methylated in a study of the effects of maternal psychosocial stress (Schroeder *et al.* 2012).

The significant weaning and MIA effects detected for PYURF are consistent with reports that this gene was under-expressed in the amygdala of low-novelty responder rats modeling anxiety-like behaviors relative to high novelty responders (Cohen 2017). Similarly, PYURF was under-expressed in weaned relative to nursed MIA pigs, while the expression was not affected in non-MIA pigs. Weaned MIA pigs showed a PYURF profile akin to anxiety-responsive rats.

Several genes presented significant simultaneous interactions of sex with weaning and sex with MIA (Table 2), including genes annotated to immune and neurological functions such as CGA, GH1, SIX1. GH1 and CGA were under-expressed in weaned relative to nursed MIA pigs and reversed and greater differences were detected among non-MIA pigs. Consistent with this finding, CGA and GH1 were under-expressed in the prefrontal cortex and cerebellum of rats that exhibited disrupted depressive-like behavior (Yamamoto *et al.* 2015).

The expression profile of SIX1 suggests that MIA reduced the effect of weaning, while this gene is substantially over-expressed in non-MIA weaned relative to nursed pigs. The transcription factor SIX1 was annotated to a cluster of over-expressed genes in high-anxiety mice exposed to LPS immune challenge (Li *et al.* 2014).

Many genes that presented significant MIA effects, alone or interacting with sex (Table 2) such as NTS, CALB1, Purkinje cell protein 4 (PCP4), and LHX9 also have neurological functions. These genes shared the pattern of under-expression in MIA relative to non-MIA pigs with some genes presenting significant interactions between MIA and sex. NTS presented opposite profiles across sexes characterized by lower NTS levels in MIA relative to non-MIA males. This profile is consistent with the lower level of NTS in the amygdala of male rats that showed lower conditioned place preference consistent with MIA-associated behaviors (Laszlo *et al.* 2010). CALB1 has been associated with regulation of synaptic plasticity and ASD (Carter 2019). PCP4-null mice presented disruptions in cerebellar synaptic plasticity and impaired locomotor learning (Wei *et al.* 2011). LHX9 presented a highly significant MIA-by-sex interaction effect that could be linked to the role of LHX9 in the sexual dimorphism such that LHX9^−/−^ mice do not produce gonads, and males display females phenotypes (Birk *et al.* 2000). Our results highlight the insights gained from studying the effect at the molecular level of postnatal stress conditional on prenatal stress and sex.

## Conclusions

The study of the expression pattern of more than 17,000 genes in the amygdalae of 22-day-old pigs uncovered evidence of disrupted immune pathways suggesting that pigs exposed to MIA are primed to be more sensitive to weaning. Also dysregulated were genes annotated to pathways with known connections to neurological disorders, including SSD and AD. The visualization of expression profiles in gene networks and study of the potential role of transcription factors such as SIX1 and ZBTB4 as possible mediators of stress within the amygdala. Additionally, the functional enrichment of pathways associated with the sex-dependent response to weaning offered a potential means by which females may be more tolerant to the adverse effects of weaning stress.

The enriched pathways and transcription factors offer possible targets for therapies that may reduce the cumulative effects of both stressors. Furthermore, identifying potentially protective pathways in females exposed to weaning stress may help develop strategies to mitigate the more adverse effects of weaning on the amygdala molecular pathways of males.

## Funding

This study is supported by USDA NIFA AFRI (grant no. 2018-67015-27413) and NIH NIDA (award no. P30 DA018310).

## Conflicts of interest

None declared.
